# Sulfatide Recognition by Colonization Factor Antigen CS6 from Enterotoxigenic *Escherichia coli*

**DOI:** 10.1371/journal.pone.0004487

**Published:** 2009-02-16

**Authors:** Lena Jansson, Joshua Tobias, Catharina Jarefjäll, Michael Lebens, Ann-Mari Svennerholm, Susann Teneberg

**Affiliations:** 1 Institute of Biomedicine, Department of Medical Biochemistry and Cell Biology, University of Gothenburg, Göteborg, Sweden; 2 Institute of Biomedicine, Department of Medical Microbiology and Immunology, University of Gothenburg, Göteborg, Sweden; University of Liverpool, United Kingdom

## Abstract

The first step in the pathogenesis of enterotoxigenic *Escherichia coli* (ETEC) infections is adhesion of the bacterium to the small intestinal epithelium. Adhesion of ETEC is mediated by a number of antigenically distinct colonization factors, and among these, one of the most commonly detected is the non-fimbrial adhesin coli surface antigen 6 (CS6). The potential carbohydrate recognition by CS6 was investigated by binding of recombinant CS6-expressing *E. coli* and purified CS6 protein to a large number of variant glycosphingolipids separated on thin-layer chromatograms. Thereby, a highly specific binding of the CS6-expressing *E. coli*, and the purified CS6 protein, to sulfatide (SO_3_-3Galβ1Cer) was obtained. The binding of the CS6 protein and CS6-expressing bacteria to sulfatide was inhibited by dextran sulfate, but not by dextran, heparin, galactose 4-sulfate or galactose 6-sulfate. When using recombinantly expressed and purified CssA and CssB subunits of the CS6 complex, sulfatide binding was obtained with the CssB subunit, demonstrating that the glycosphingolipid binding capacity of CS6 resides within this subunit. CS6-binding sulfatide was present in the small intestine of species susceptible to CS6-mediated infection, *e.g.* humans and rabbits, but lacking in species not affected by CS6 ETEC, *e.g.* mice. The ability of CS6-expressing ETEC to adhere to sulfatide in target small intestinal epithelium may thus contribute to virulence.

## Introduction

Attachment of microbes to cell surface receptors on the target tissue is considered an essential step in the initiation, establishment and maintenance of infection. As a result great interest has been shown in the elucidation and identification of potential microbial host receptors, the majority of which appear to be glycoconjugates [1]–[3]. Glycoconjugates exhibit a characteristic and specific pattern of expression, which is dependent on the animal species, individual and cell type [4], thus explaining the phenomenon of tropism of infection.

Adherence of enterotoxigenic *Escherichia coli* (ETEC) is mediated by colonization factors (CFs), which usually are fimbrial structures present on the bacterial cell surface. Infecting ETEC adhere to and colonize the intestinal epithelium, and cause diarrhea primarily by the production of heat-labile and/or heat-stable enterotoxin (LT and ST respectively). Around 25 different CFs have been identified [5], and one of the most commonly detected is coli surface antigen 6 (CS6) [5]–[8]. CS6 is non-fimbrial, but the overall structure of this adhesin has not yet been defined.

The CS6 operon required for assembly of the CS6 adhesin contains four open reading frames [9]. Two heterologous major structural subunits, CssA and CssB, are encoded by the *cssA* gene and the *cssB* gene, respectively. The *cssC* gene encodes a chaperone that was assumed to assist in the folding of CssA and CssB, and *cssD* encodes a tentative usher involved in the transport of CssA and CssB across the outer membrane. In a recent study a series of deletions were made in each of the genes of the CS6 operon, and the effects on the expression of CssA and CssB were examined [10]. Deletion of the chaperone CssC gave reduced levels of the CssA subunit, while the expression of the CssB subunit was not affected. Reduced levels of the CssA subunit was also obtained when the *cssB* gene was deleted. Thus, the CssA subunit requires CssC for folding, and is stabilized by interaction with CssB. Surprisingly, the expression of the CssA and CssB subunits was not affected by deletion of the usher CssD, leading to the suggestion that the usher is not involved in the assembly or surface expression of CS6.

Using the rabbit non-ligated intestinal model (RITARD) it has been shown that CS6 mediates binding of ETEC in rabbit intestine, where colonization was obtained by a CS6-positive strain, but not with the isogenic CS6-deficient strain [11]. A previous study has also shown that the binding of CS6 to rabbit enterocytes, and to mucus from rabbit and human intestines, was abolished by treatment with meta-periodate, indicating the involvement of carbohydrates in the binding process [12].

To further examine the potential role of carbohydrates as adhesion receptors for CS6, the binding of purified CS6 protein and recombinant CS6-expressing *E. coli* to glycosphingolipids was investigated in the present study. Thereby, a specific interaction between CS6-expressing bacteria, and purified CS6 protein, and sulfatide (SO_3_-3Galβ1Cer) was detected. Binding assays with purified CssA and CssB subunits demonstrated that the CssB subunit carries the sulfatide binding capacity. Furthermore, a correlation between the expression of sulfatide in target cells and susceptibility to CS6-mediated ETEC infection was found.

## Results

### Characterization of the CS6 protein and the CssA and CssB subunits

**Figure 1:**
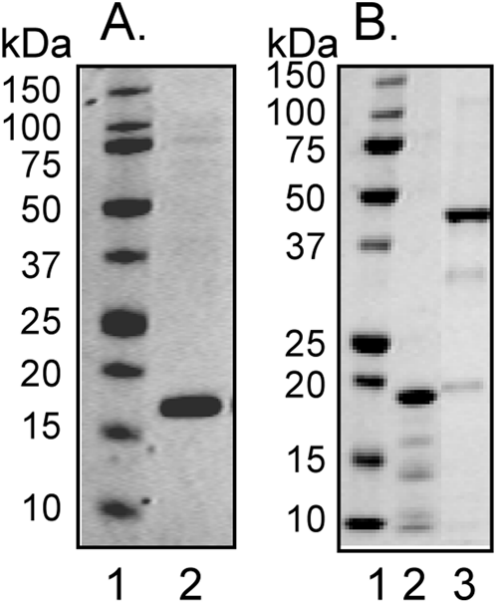
Purified recombinant CS6, CssA and CssB proteins. The protein preparations were separated on 10% (A) and 12% (B) NuPAGE BisTris gels, and stained by Coomassie Brilliant Blue R-250. The lanes on A were 1, molecular mass standards; 2, CS6 protein, and the lanes on B were 1, molecular mass standards; 2, CssA protein (with polyhistidine tag); 3, CssB protein (fused to glutathione-S-transferase (26 kDa) and with polyhistidine tag).

The three proteins were purified by the chromatgraphic steps described in the Materials and methods section. The purified proteins migrated as single bands, and the apparent molecular weights were in agreement with the predicted molecular masses for the constructed proteins 18483.61 for CssA (with the polyhistidine tag) and 43,997.93 for CssB (fused to glutathione-S-transferase carrying a His tag (Fig. 1).

### Specific recognition of sulfatide by CS6-expressing *E. coli* and the CS6 protein

**Figure 2:**
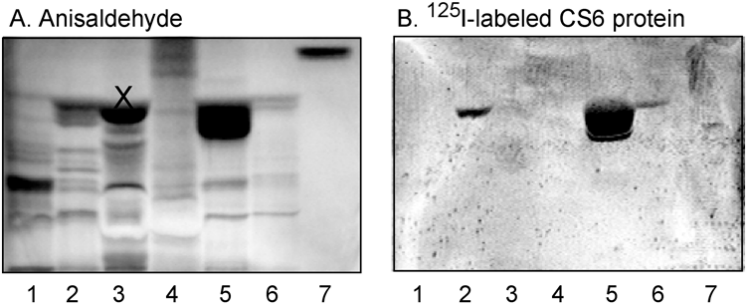
Binding of ^125^I-labeled CS6 adhesin to mixtures of glycosphingolipids on thin-layer chromatograms. Chemical detection by anisaldehyde (A), and autoradiogram obtained by binding of ^125^I-labeled CS6 protein (B). The glycosphingolipids were separated on aluminum-backed silica gel plates, using chloroform/methanol/water (60∶35∶8, by volume) as solvent system, and the binding assay was performed as described under “Materials and methods”. Autoradiography was for 12 h. The lanes were: Lane 1, acid glycosphingolipids of human hepatoma, 40 µg; Lane 2, acid glycosphingolipids of human small intestine, 40 µg; Lane 3, acid glycosphingolipids of guinea pig erythrocytes, 40 µg; Lane 4, acid glycosphingolipids of guinea pig stomach, 40 µg; Lane 5, acid glycosphingolipids of human meconium, 40 µg; Lane 6, acid glycosphingolipids of human colon cancer, 40 µg; Lane 7, glucosylceramide (Glcβ1Cer), 4 µg. The band in marked with an X in lane 3 was stained blue by anisaldehyde, and thus a non-glycosphingolipid contaminant [36].

In order to expose the CS6 protein to a large number of potentially binding-active carbohydrate structures, mixtures of glycosphingolipids from various sources separated on thin-layer plates were used in the initial screening for carbohydrate recognition by the CS6 adhesin. When using mixtures of acid glycosphingolipids, *i.e.* sulfate-containing glycosphingolipids and sialic acid- containing glycosphingolipids (gangliosides), a distinct binding of the protein to a fast-migrating compound in the acid fraction of human small intestine (Fig. 2, lane 2), human meconium (lane 5), and human colon cancer (lane 6) was observed. No binding of the CS6 protein to the more slow-migrating compounds (mainly gangliosides) in the acid fractions, or to any non-acid glycosphingolipids was obtained.

The binding of the CS6 protein to a fast-migrating compound in the acid fraction of human small intestine and human meconium directed our interest towards sulfate-containing glycosphingolipids, since the major glycosphingolipid of these fractions is sulfatide [13], [14].

**Figure 3:**
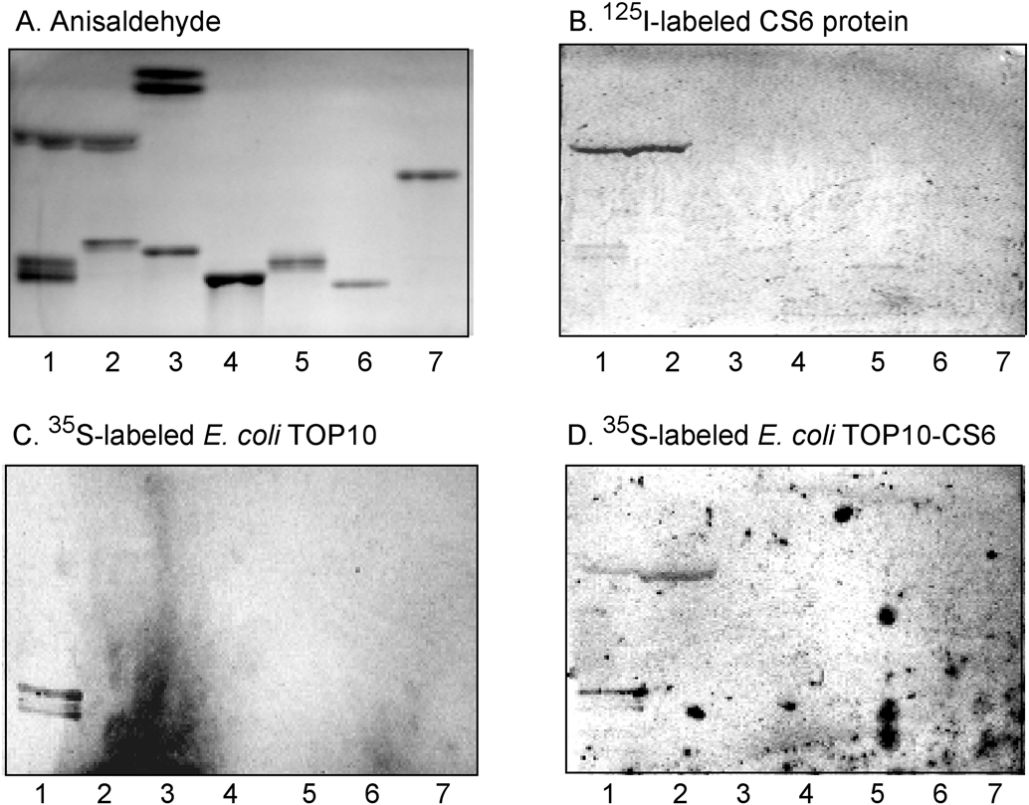
Binding of CS6 protein, vector *E. coli* TOP10 strain, and recombinant *E. coli* TOP10-CS6 strain to pure glycosphingolipids on thin-layer chromatograms. Chemical detection by anisaldehyde (A), and autoradiograms obtained by binding of ^125^I-labeled CS6 protein (B), ^35^S-labeled *E. coli* TOP10 strain (C) and ^35^S-labeled *E. coli* TOP10-CS6 strain (D). The glycosphingolipids were separated on aluminum-backed silica gel plates, using chloroform/methanol/water (60∶35∶8, by volume) as solvent system, and the binding assays were performed as described under “Materials and methods”. Autoradiography was for 12 h. The lanes were: Lane 1, sulfatide (SO_3_-3Galβ1Cer), 4 µg, and fucosyl-gangliotetraosylceramide (Fucα2Galβ3GalNAcβ4Galβ4Glcβ1Cer), 4 µg; Lane 2, sulfatide (SO_3_-3Galβ1Cer), 4 µg, and Forssman glycosphingolipid (GalNAcα3GalNAcβ3Galα4Galβ4Glcβ1Cer), 4 µg; Lane 3, galactosylceramide (Galβ1Cer), 4 µg, and blood group H type 2 pentaglycosylceramide (Fucα2Galβ4GlcNAcβ3Galβ4Glcβ1Cer), 4 µg; Lane 4, blood group B type 2 hexaglycosylceramide (Galα3(Fucα2)Galβ4GlcNAcβ3Galβ4Glcβ1Cer), 4 µg; Lane 5, blood group A type 2 hexaglycosylceramide (GalNAcα3(Fucα2)Galβ4GlcNAcβ3Galβ4Glcβ1Cer), 4 µg; Lane 6, blood group A type 2 heptaglycosylceramide (GalNAcα3(Fucα2)Galβ4(Fucα3)GlcNAcβ3Galβ4Glcβ1Cer), 4 µg; Lane 7, globotriaosylceramide, (Galα4Galβ4Glcβ1Cer), 4 µg.

Next the binding of the CS6 protein, the recombinant CS6-expressing *E. coli* strain TOP10-CS6, and the *E. coli* TOP10 background strain to a number of pure glycosphingolipids at defined concentrations was tested in the chromatogram binding assay. The results are exemplified in Fig. 3, and summarized in Table 1. The majority of the glycosphingolipids tested, *i.e.* the non-acid glycosphingolipids (Nos. 1,2,4,6-8, 10-21) and the gangliosides (Nos. 22-27), were not recognized by the CS6 protein or the CS6-expressing bacteria. However, both the CS6 protein and the CS6-positive *E. coli*, but not the TOP10 vector strain, bound to sulfatide (SO_3_-3Galβ1Cer; No. 3 in Table 1; Fig. 3B and D, lanes 1 and 2, top band) and to sulfo-lactosylceramide (SO_3_-Galβ4Glcβ1Cer; No. 5 in Table 1). No binding to galactosylceramide (Galβ1Cer; No. 1 in Table 1; Fig. 2, lane 3, top band,) or to lactosylceramide (Galβ4Glcβ1Cer; No. 4 in Table 1) was obtained, demonstrating that the sulfate group of sulfatide was necessary for the interaction. However, sulfo-gangliotetraosylceramide (SO_3_-3Galβ3GalNAcβ4Galβ4Glcβ1Cer; No. 9 in Table 1) was not recognized by the CS6 protein or CS6-positive bacteria.

**Table 1:** Binding of ^125^I-labeled native CS6 protein, ^35^S-labeled recombinant CS6-expressing *Escherichia coli* (TOP10-CS6) and ^35^S-labeled background *E. coli* strain TOP10 to glycosphingolipids on thin-layer chromatograms.

No. Trivial name	Structure	CS6 protein	TOP10-CS6	TOP 10
*Simple compounds*				
1. Galactosylceramide	Galβ1Cer	− a	−	−
2. Glucosylceramide	Glcβ1Cer	−	−	−
3. Sulfatide	SO_3_-3Galβ1Cer	+	+	−
4. LacCer	Galβ4Glcβ1Cer	−	−	−
5. Sulf-LacCer	SO_3_-3Galβ4Glcβ1Cer	+	+	−
6. Globotri	Galα4Galβ4Glcβ1Cer	−	−	−
*Ganglioseries*				
7. GgO3	GalNAcβ4Galβ4Glcβ1Cer	−	−	−
8. Fuc-GgO4	Fucα2Galβ3GalNAcβ4Galβ4Glcβ1Cer	−	+	+
9. Sulf-GgO4	SO_3_-3Galβ3GalNAcβ4Galβ4Glcβ1Cer	−	−	−
*Neolactoseries*				
10. Neolactotetra	Galβ4GlcNAcβ3Galβ4Glcβ1Cer	−	−	−
11. H5-2	Fucα2Galβ4GlcNAcβ3Galβ4Glcβ1Cer	−	−	−
12. B6-2	Galα3(Fucα2)Galβ4GlcNAcβ3Galβ4Glcβ1Cer	−	−	−
13. A6-2	GalNAcα3(Fucα2)Galβ4GlcNAcβ3Galβ4Glcβ1Cer	−	−	−
14. A7-2	GalNAcα3(Fucα2)Galβ4(Fucα3)GlcNAcβ3Galβ4Glcβ1Cer	−	−	−
*Lactoseries*				
15. Le^a^-5	Galβ3(Fucα4)GlcNAcβ3Galβ4Glcβ1Cer	−	−	−
16. Le^b^-6	Fucα2Galβ3(Fucα4)GlcNAcβ3Galβ4Glcβ1Cer	−	−	−
17. A7-1	GalNAcα3(Fucα2)Galβ3(Fucα4)GlcNAcβ3Galβ4Glcβ1Cer	−	−	−
18. B7-1	Galα3(Fucα2)Galβ3(Fucα4)GlcNAcβ3Galβ4Glcβ1Cer	−	−	−
*Globoseries*				
19. Globotetra	GalNAcβ3Galα4Galβ4Glcβ1Cer	−	−	−
20. Isoglobotetra	GalNAcβ3Galα3Galβ4Glcβ1Cer	−	−	−
21. Forssman	GalNAcα3GalNAcβ3Galα4Galβ4Glcβ1Cer	−	−	−
*Gangliosides*				
22. GM3	NeuAcα3Galβ4Glcβ1Cer	−	−	−
23. GD3	NeuAcα8NeuAcα3Galβ4Glcβ1Cer	−	−	−
24. GM1	Galβ3GalNAcβ4(NeuAcα3)Galβ4Glcβ1Cer	−	−	−
25. GD1a	NeuAcα3Galβ3GalNAcβ4(NeuAcα3)Galβ4Glcβ1Cer	−	−	−
26. NeuAcα3SPG	NeuAcα3Galβ4GlcNAcβ3Galβ4Glcβ1Cer	−	−	−

a Binding is defined as follows: + denotes a binding when 4 µg of the glycosphingolipid was applied on the thin-layer chromatogram, while − denotes no binding even at 4 µg.

The TOP10 background strain binds to glycosphingolipids of the ganglio-series, as *e.g.* fucosyl-gangliotetraosylceramide (Fucα2Galβ3GalNAcβ4Galβ4Glcβ1Cer; No. 8 in Table 1; Fig. 3C, lane 1, lower band) [15], and this binding was also obtained with the recombinant TOP10-CS6 strain (Fig. 3D). However, while both the CS6 protein and the CS6-positive *E. coli* bound to sulfatide, no binding of the TOP10 background strain to this compound was obtained, demonstrating that the recognition of sulfatide by the TOP10-CS6 strain was indeed dependent on the CS6 protein.

**Figure 4:**
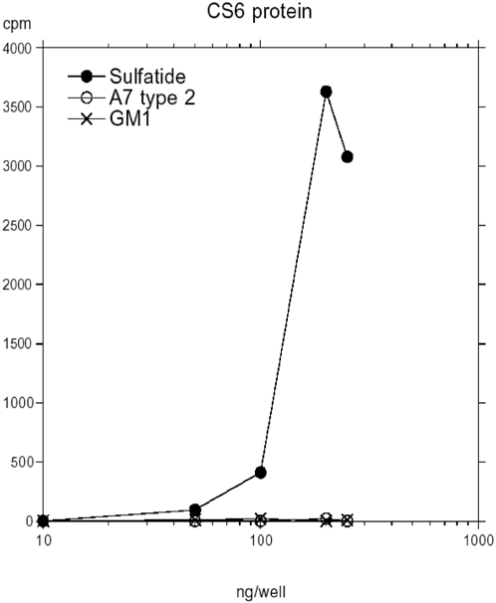
Binding of ^125^I-labeled CS6 protein to serial dilutions of pure glycosphingolipids in microtiter wells. The assay was performed as described in the Materials and methods section. The results from one representative experiment out of three is shown.

The binding of the CS6 protein to glycosphingolipids was also tested in the microtiter well assay. Also in this assay system sulfatide was the preferred ligand (half-maximal binding at approximately 100 ng), while other glycosphingolipids tested, as the A7 type 2 glycosphingolipid (GalNAcα3(Fucα2)Galβ4(Fucα3)GlcNAcβ3Galβ4Glcβ1Cer) and the GM1 ganglioside (Galβ3GalNAcβ4(NeuAcα3)Galβ4Glcβ1Cer) gave no signal (Fig. 4).

### Sulfatide binding of the CS6 protein and CS6-expressing *E. coli* is inhibited by dextran sulfate

**Figure 5:**
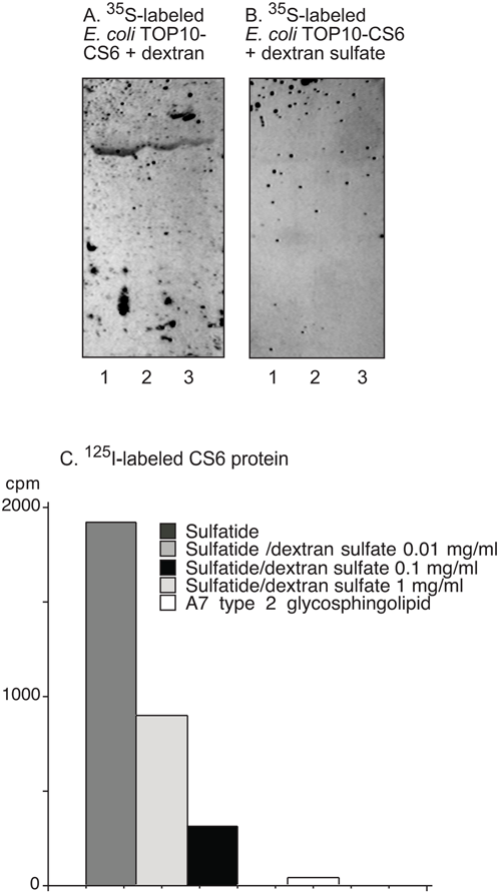
Effects of incubation of CS6 protein and recombinant CS6-expressing *E. coli* with dextran and dextran sulfate. Radiolabeled CS6 protein and recombinant CS6-expressing bacteria were incubated with dextran or dextran sulfate (0.01–1 mg/ml) for 1 h at room temperature. Thereafter the suspensions were utilized in the chromatogram binding assay or the microtiter well assay as described under “Materials and Methods”. Autoradiograms obtained by binding of ^35^S-labeled *E. coli* TOP10-CS6 strain incubated with dextran (A), and with dextran sulfate (B). Autoradiography was for 12 h. The lanes were: Lane 1, sulfatide (SO_3_-3Galβ1Cer), 4 µg; Lane 2, sulfatide, 2 µg; Lane 3, sulfatide, 1 µg. Binding of ^125^I-labeled CS6 protein, and ^125^I-labeled CS6 protein incubated with dextran sulfate, to pure glycosphingolipids in microtiter wells (C).

The ability of sulfated carbohydrates to interfere with the binding of the CS6 adhesin to glycosphingolipids was examined by incubating the CS6 protein, and CS6-expressing *E. coli*, with dextran, dextran sulfate, heparin, galactose 4-sulfate, or galactose 6-sulfate before binding to glycosphingolipids on chromatograms or in microtiter wells. As shown in Fig. 5, incubation with dextran sulfate (1 mg/ml) ablated the sulfatide binding of the CS6-expressing bacteria on thin-layer chromatograms (Fig. 5B). Also the binding of the CS6 protein to sulfatide was reduced in a dose-dependent manner by incubation with dextran sulfate (Fig. 5C). In contrast, incubation with dextran (Fig. 5A), heparin, galactose 4-sulfate, or galactose 6-sulfate (each 1 mg/ml) (not shown) did not affect the binding of CS6-positive *E. coli*, or the CS6 protein, to sulfatide.

### Sulfatide binding is mediated by the CssB subunit

**Figure 6:**
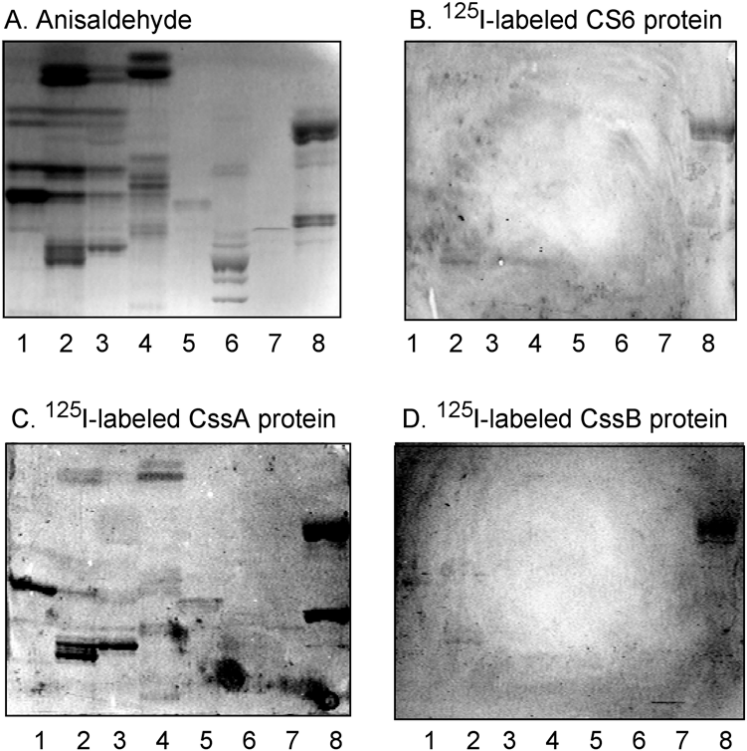
Binding of ^125^I-labeled CS6 protein, and CssA and CssB subunits, to mixtures of glycosphingolipids on thin-layer chromatograms. Chemical detection by anisaldehyde (A), and autoradiograms obtained by binding of ^125^I-labeled CS6 (B), CssA (C) and CssB (D). The glycosphingolipids were separated on aluminum-backed silica gel plates, using chloroform/methanol/water (60∶35∶8, by volume) as solvent system, and the binding assay was performed as described under “Materials and methods”. The lanes were: Lane 1, non-acid glycosphingolipids of human erythrocytes, 40 µg; Lane 2, non-acid glycosphingolipids of human small intestine (Individual No. 1), 40 µg; Lane 3, non-acid glycosphingolipids of human small intestine (Individual No. 2), 40 µg; Lane 4, non-acid glycosphingolipids of rat intestine, 40 µg; Lane 5, non-acid glycosphingolipids of human meconium, 40 µg; Lane 6, calf brain gangliosides, 40 µg; Lane 7, acid glycosphingolipids of human erythrocytes, 40 µg; Lane 8, acid glycosphingolipids of piglet intestine, 40 µg. Autoradiography was for 12 h.

In order to evaluate the roles of the CssA and CssB structural subunits of CS6 in the sulfatide binding process, these two proteins were expressed separately. Chromatogram binding experiments using ^125^I-labeled subunits gave a specific binding of the CssB subunit to sulfatide, the major acid glycosphingolipid of piglet intestine [16] (Fig. 6D, lane 8), in the same manner as the CS6 protein (Fig. 6B, lane 8). The CssA subunit, on the other hand, bound to several major compounds both in the non-acid fractions (Fig. 6C, lanes 1–5) and in the acid fractions (Fig. 6C, lanes 6–8), in an unspecific manner demonstrating a general stickiness of this protein in the absence of CssB. Thus, the structural element of the CS6 adhesin responsible for sulfatide recognition resides within the CssB subunit.

### Presence of sulfatide in target small intestinal target cells

**Figure 7:**
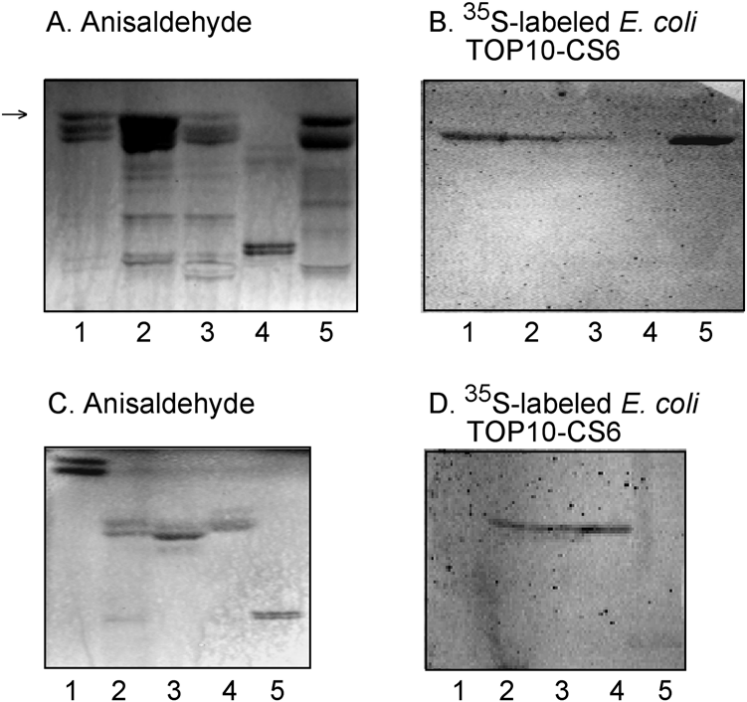
Binding of recombinant CS6-expressing *E. coli* to intestinal acid glycosphingolipids on thin-layer chromatograms. Chemical detection by anisaldehyde (A and C), and autoradiograms obtained by binding of ^35^S-labeled CS6-expressing *E. coli* (TOP10-CS6) (B and D). The glycosphingolipids were separated on aluminum-backed silica gel plates, using chloroform/methanol/water (60∶35∶8, by volume) as solvent system, and the binding assays were performed as described under “Materials and methods”. Autoradiography was for 12 h. The lanes on A and B were: Lane 1, acid glycosphingolipids of human small intestine (Individual No. 1), 40 µg; Lane 2, acid glycosphingolipids of human small intestine (Individual No. 2), 40 µg; Lane 3, acid glycosphingolipids of human small intestine (Individual No. 3), 40 µg; Lane 4, acid glycosphingolipids of mouse small intestine, 40 µg; Lane 5, acid glycosphingolipids of rabbit small intestine, 40 µg. The arrow denotes a band stained blue by anisaldehyde, and thus a non-glycosphingolipid contaminant [36]. The lanes on C and D were: Lane 1, galactosylceramide (Galβ1Cer), 4 µg; Lane 2, acid glycosphingolipids of human small intestinal epithelium (Individual No. 4), 40 µg; Lane 3, sulfatide (SO_3_-3Galβ1Cer) with phytosphingosine and hydroxy 16∶0 fatty acid from human small intestinal epithelium, 4 µg; Lane 4, sulfatide (SO_3_-3Galβ1Cer) with phytosphingosine and hydroxy 24∶1 fatty acid from human small intestinal epithelium, 4 µg; Lane 5, sulf-gangliotetraosylceramide (SO_3_-3Galβ3GalNAcβ4Galβ4Glcβ1Cer), 4 µg.

To assess the potential role of the sulfatide recognition by CS6 in target tissue adherence, the binding of recombinant CS6-expressing *E. coli* to total acid glycosphingolipid fractions from human, mouse and rabbit small intestines was examined. The CS6-positive bacteria bound to a compound migrating as sulfatide in the acid glycosphingolipid fractions from human small intestine (Fig. 7, lanes 1–3), and rabbit small intestine (Fig. 7, lane 5), while no binding to the acid glycosphingolipids from mouse small intestine (Fig. 7, lane 4) occurred. Sulfatide isolated from human small intestine was also recognized by the CS6-expressing bacteria (Fig. 7D, lanes 3 and 4).

## Discussion

Susceptibility to CS6-mediated infections varies among species, and while *e.g.* humans and rabbits are highly susceptible, mice are not colonized [11], [17]. Here we report that the CssB subunit of the CS6 protein confers a specific binding of this colonization factor to a glycosphingolipid present in human and rabbit small intestine. This CS6-binding compound was identified as sulfatide (SO_3_-3Galβ1Cer), which is the major acid glycosphingolipid of the human small intestinal epithelial cells [13]. Binding of CS6-expressing *E. coli* to sulfatides isolated from human small intestinal epithelium was also demonstrated.

The binding studies with the recombinant CssA and CssB subunits were done with proteins having remnant affinity tags. We have now developed an affinity system based on a sulfate-carrying polymer (Lebens *et al.*, to be published). The CS6 protein and the native CssB subunit, but not the native CssA subunit, are retained on this affinity column, supporting the suggestion that the sulfatide recognition of CS6 is mediated by the CssB subunit. Furthermore, in a recent study the binding to CaCo-2 cells by recombinant *E. coli* with deletion of the *cssA* gene was reported [10]. In contrast, upon deletion of the *cssB* gene, the cell binding by the bacteria was not statistically different from that obtained with the background strain.

Although sulfatide is a common constituent among small intestinal glycosphingolipids of most species, its presence is not obligatory, since it is not found in the small intestine of *e.g.* mouse, rat, cod [18] or calves [19]. In mouse small intestinal epithelium, the major sulfo-glycosphingolipid is sulf-gangliotetraosylceramide (SO_3_-3Galβ3GalNAcβ4Galβ4Glcβ1Cer) [20], which, as we show here, is not recognized by the CS6 protein or CS6-positive *E. coli*.

A dose-dependent reduction of the CS6-mediated sulfatide binding was obtained with the highly sulfated polymer dextran sulfate, but not with dextran, demonstrating the importance of the sulfate group for binding to occur. Preincubation with heparin, galactose 4-sulfate, galactose 6-sulfate had no inhibitory effect, suggesting a specific recognition of the sulfate group in 3-position of galactose as in sulfatide, or glucose as in dextran sulfate. The recognition of sulfo-lactosylceramide (SO_3_-3Galβ4Glcβ1Cer) by CS6 protein and the CS6-expressing bacteria is in agreement with this suggestion. However, sulfo-gangliotetraosylceramide (SO_3_-3Galβ3GalNAcβ4Galβ4Glcβ1Cer) was not recognized by the CS6 protein or CS6-positive bacteria, although it has a terminal SO_3_-3Gal. Thus, a SO_3_-3Galβ3GalNAc sequence is not tolerated, suggesting a sterical hindrance caused by the *N*-acetylgalactosamine.

Phenotypic expression of CS6 is associated with expression of the heat-stable enterotoxin (ST-only and ST/LT strains), while it is rarely observed in ETEC strains expressing only the heat-labile enterotoxin [5], [21]. There are two subtypes of the heat-stable toxin, designated STa and STb. The receptor for STa, which is associated with diarrhea in humans and animals, piglets, is the extracellular domain of a trans-membrane guanylate cyclase type C protein [22]. Interestingly, the STb subtype, which is primarily associated with diarrhea in piglets, binds to sulfatide [23]. The interaction of STb with sulfatide is not affected by dextran sulfate, in contrast to the inhibitory effect observed for the sulfatide binding of the CS6 protein.

Sulfatide recognition has also been reported for other pathogens, including *Mycoplasma pneumoniae* [24], *Bordetella pertussis* [25], 987P-fimbriated *E.coli* [26], and *Helicobacter pylori* [27], [28]. In the case of *H. pylori* the heat-shock protein Hsp 70 has been proposed to be the adhesin involved in sulfatide binding [29], [30]. In addition, sulfatide is recognized by HP-NAP, the soluble neutrophil-activating protein of *H. pylori* [31].

The sulfatide binding of CS6-expressing ETEC differs distinctly from the glycosphingolipid specificity of CFA/I-fimbriated ETEC [15]. The CFA/I minor tip subunit CfaE mediates hemagglutination and binding to CaCo-2 cells by binding to an as yet unidentified receptor [32]. The major CfaB subunit of CFA/I binds to a number of non-acid glycosphingolipids, including glucosylceramide, lactosylceramide with phytosphingosine and/or hydroxy fatty acids, neolactotetraosylceramide, gangliotriaosylceramide, gangliotetraosylceramide, the H5 type 2 pentaglycosylceramide, the Le^a^-5 glycosphingolipid, the Le^x^-5 glycosphingolipid and the Le^y^-6 glycosphingolipid. These non-acid glycosphingolipids were also recognized by fimbriae with high sequence similarity to CFA/I, *i.e.* CS1 and CS4 [12], but not by the CS6 adhesin (present study). However, there is a large repertoire of CFs expressed by ETEC, and for the major part of these CFs the attachment sites are not yet defined. The mechanisms of adherence mediated by ETEC CFs thus merits further studies.

## Materials and Methods

### Bacterial strains, culture and labeling

Recombinant CS6 adhesin was expressed in the *E. coli* strain TOP10 as described by Tobias *et al.* [10], and the resulting recombinant strain was designated TOP10-CS6. The TOP10-CS6 strain was grown in CFA broth containing Casamino acids 10 g, yeast extract 1.5 g, MgSO_4_.7H_2_O 102 mg, and MnCl_2_.4H_2_O 8 mg per liter (pH 7.4), supplemented with ampicillin (100 µg/ml) at 37°C overnight with shaking. For metabolic labeling, 100 µl of this bacterial suspension was transferred to 10 ml of the same medium supplemented with 10 µl ^35^S-methionine (400 µCi; Amersham Pharmacia Biotech), and grown for 2 h at 37°C with shaking. Thereafter, isopropyl-β-D-thiogalactopyranoside (IPTG; Saveen Werner AB, Malmö, Sweden) to the final concentration of 1 mM was added, and the bacteria were then incubated overnight. Bacteria were harvested, washed three times in phosphate-buffered saline (PBS, pH 7.3), then resuspended in PBS) containing 2% (w/v) bovine serum albumin, 0.1% (w/v) NaN_3_ and 0.1% (w/v) Tween 20 (BSA/PBS/TWEEN) to a bacterial density of 1×10^8^ colony forming units/ml. The specific activity of bacterial suspensions was approximately 1 cpm per 100 bacteria.

The same conditions (with omission of ampicillin and IPTG) were used for culture and labeling of the background *E. coli* strain TOP10.

### Isolation of CS6 protein

The CS6 protein was isolated from the TOP10-CS6 recombinant strain. An overnight culture of TOP10-CS6, grown in CFA broth supplemented with 100 µg/ml of ampicillin, was diluted 1/100 in the same medium and incubated for 2 h at 37°C and 150 rev/min. IPTG was then added into the medium to the final concentration of 1 mM, and the bacteria were grown at 37°C and 150 rpm for 17 h. The bacterial culture was centrifuged twice at 10000×g for 15 min. The supernatant was then filtered (0.2 µm Amicon; Millipore, Bedford, MA) to remove bacterial cells. The supernatant containing the CS6 was subjected to overnight ammonium sulfate precipitation (50% saturation) at 4°C, and the protein was recovered by centrifugation at 13000×g for 30 min. The protein was dialyzed extensively against PBS and stored at −70°C.

### Production and purification of CssA and CssB

DNA fragments encoding the mature CssA and CssB proteins were each amplified from the recombinant plasmid pJT-CS6 [10]. Each fragment was amplified so that it could be inserted directly into an appropriate expression vector. The primers for the amplification were:

*cssA*
Forward: 5′-CCCCGGTCTCACAAGAGAACAGAAATAGCGACTAAAAACTTC-3′
Reverse: 5′-CCCCGGTACCTTAATTAATTAGTTTACATAGTAACCAACCATAACC-3′
*cssB*
Forward: 5′-CCCCGGTCTCACAAGGGAAACTGGCAATATAAATCTCTGGATG-3′
Reverse: 5′-CGGTCTCAAGCTTAATTGCTGTAAAATGATACAGTCAAATG-3′

The amplified *cssA* fragment contained terminal BsaI and KpnI sites that allowed directional cloning into a modified pBAD/His plasmid [10] such that the resulting protein carried an amino terminus polyhistidine tag that could be removed by enterokinase digestion resulting in the native CssA protein. Protein expression was induced by growth of the recombinant TOP10 strain in the presence of 0.1% arabinose. This resulted in the accumulation of the recombinant protein as cytoplasmic inclusion bodies, since the signal peptide required for transport through the inner membrane to the periplasm had been removed. The cells were harvested by centrifugation and disrupted by lysozyme and DNase I treatment followed by sonication. The inclusion bodies were separated from the other cell debris by low speed centrifugation. Following extensive washing of the inclusion bodies they were finally dissolved in 6.5 M urea. The urea was removed by dialysis against 20 mM phosphate buffer, pH 7.4. When the urea had been removed the his tagged protein was purified by affinity chromatography using a nickel loaded His trap column (Amersham Pharmacia Biotech, Uppsala, Sweden) attached to an ÄKTA FPLC apparatus (Pharmacia). The protein was loaded onto the column in 20 mM phosphate buffer, pH 7.4, made 10 mM with respect to imidazole, essentially according to the instructions of the manufacturers. The protein was eluted using an imidazole gradient from 10 mM up to 500 mM. The peak containing the recombinant protein was identified by SDS-PAGE electrophoresis. Fractions containing the protein were pooled and dialyzed against 20 mM phosphate buffer, pH 7.4, in order to remove the imidazole. Purity of the resulting CssA product was estimated from SDS-PAGE to be greater than 95%. Protein concentration was determined by a modified Biuret assay.

Following cloning, CssB was not successfully expressed in the same system. However, the mature CssB protein was also fused to the 26 kDa glutathione-S-transferase (sj26GST) from *Schistosoma japonicum* using an expression vector constructed in this laboratory. The fusion protein was constructed such that cssB protein could be cleaved from sj26GST carrier in two ways. Using thrombin for cleavage, the CssB carries an amino terminus extension containing a polyhistidine tag. Cleavage with enterokinase, however, leads to removal of the native CssB protein from the sj26GST, carrier which retains the polyhistidine tag. This allows the carrier protein to be removed from the CssB using a chelating column charged with nickel ions, even if the glutathione-S-transferase (GST) is not active and cannot act as an affinity ligand. A strain carrying the recombinant plasmid was grown up under inducing conditions in which IPTG was added to the growth medium to a final concentration of 1 mM. As with CssA, recombinant protein accumulated in the cytoplasm of the bacteria as insoluble inclusion bodies. The inclusion bodies were isolated and dissolved as described above, and treated in exactly the same way as CssA, taking advantage of the polyhistidine tag and avoiding difficulties related to the reconstitution of active GST.

### Electrophoresis

The protein preparations were separated on 10% or 12% NuPAGE BisTris gels (Life Technologies Inc., Carlsbad, CA) according to the instructions of the manufacturer, and stained by Coomassie Brilliant Blue R-250.

### ^125^I-labeling

Aliquots of 100 µg of protein were labeled with ^125^I, using Na^125^I (100 µCi/ml; Amersham Pharmacia Biotech, Little Chalfont, U.K.), according to the IODO-GEN protocol of the manufacturer (Pierce, Rockford, IL), giving approximately 5×10^3^ cpm/µg protein.

### Reference glycosphingolipids

Total acid and non-acid glycosphingolipid fractions were prepared as described earlier [33]. Individual glycosphingolipids were isolated by repeated chromatography on silicic acid columns and by HPLC, and identified by mass spectrometry [34], and ^1^H-NMR spectroscopy [35].

### Thin-layer chromatography

Aluminum- or glass-backed silica gel 60 high performance thin-layer chromatography plates (Merck, Darmstadt, Germany) were used for thin-layer chromatography, and eluted with chloroform/methanol/water (60∶35∶8, by volume) as solvent system. The different glycosphingolipids were applied to the plates in quantities of 1–4 µg of pure glycosphingolipids and 40 µg of glycosphingolipid mixtures. Chemical detection was done with anisaldehyde [36].

### Chromatogram binding assay

Binding of ^125^I-labeled CS6 protein, CssA and CssB subunits, and of ^35^S-labeled *E. coli* to glycosphingolipids on thin-layer chromatograms was done as described previously [15]. Dried chromatograms were dipped in diethylether/*n*-hexane (1∶5 v/v) containing 0.5% (w/v) polyisobutylmethacrylate for 1 min, dried, and then blocked with BSA/PBS/TWEEN for 2 h at room temperature. Thereafter the plates were incubated with ^125^I-labeled CS6, CssA or CssB protein (1–5×10^6^ cpm/ml), or ^35^S-labeled bacteria (1–5×10^6^ cpm/ml), diluted in BSA/PBS/TWEEN for another 2 h at room temperature. After washing six times with PBS, and drying, the thin-layer plates were autoradiographed for 12 h using XAR-5 x-ray films (Eastman Kodak, Rochester, NY).

### Microtiter well binding assay

Binding of ^125^I-labeled CS6 protein to glycosphingolipids coated in microtiter wells was performed as previously described [31]. In short, serial dilutions (each dilution in triplicate) of pure glycosphingolipids in methanol were applied in microtiter wells (Falcon 3911, Becton Dickinson Labware, Oxnard, CA). When the solvent had evaporated, the wells were blocked for 2 h with 200 µl of BSA/PBS/TWEEN. Thereafter, 50 µl of radiolabeled CS6 protein diluted in BSA/PBS/TWEEN (approximately 2×10^3^ cpm/µl), were added per well and incubated for 4 h at room temperature. After washing 6 times with PBS, the wells were cut out and the radioactivity counted in a gamma counter.

### Inhibition studies

As a test for possible inhibition of binding, ^125^I-labeled CS6 protein or ^35^S-labeled *E. coli* Top10-CS6 were incubated with various concentrations (1 mg/ml, 0.1 mg/ml and 0.01 mg/ml) of saccharides and anionic polysaccharides in PBS. Incubations were done for 2 h at room temperature, and thereafter the suspensions were diluted 40 times, and used in the chromatogram binding assay (the ^35^S-labeled CS6-positive bacteria), or the microtiter well assay (the ^125^I-labeled CS6 protein), as described above. Dextran sulfate, dextran, and heparin were from VWR International AB., Stockholm, while galactose 4-sulfate, and galactose 6-sulfate were from Sigma, St. Louis, MO.

### Isolation and characterization of sulfatides from human small intestinal epithelium

Total acid glycosphingolipids were isolated from mucosal scrapings of the small intestine of one single individual as described [33]. Part of the total acid fraction (13.3 mg) was separated on an Iatrobeads (Iatrobeads 6RS-8060; Iatron Laboratories, Tokyo) column (10 g), eluted with chloroform/methanol/water 65∶25∶4 (by volume), 10×5 ml, followed by chloroform/methanol/water 40∶40∶12 (by volume), 2×10 ml. Aliquots of the fractions that were coloured by anisaldehyde on thin-layer plates were tested for binding of the CS6 protein using the chromatogram binding assay. Binding of the CS6 protein to compounds migrating as reference sulfatide was obtained in four consecutive fractions (fractions 4–7), and these fractions were characterized by negative ion FAB mass spectrometry using a JEOL SX-102A mass spectrometer (JEOL, Tokyo, Japan). The ions were produced by 6 keV xenon atom bombardment, using triethanolamine (Fluka, Buchs, Switzerland) as matrix, and an accelerating voltage of −10 kV. Sulfatide with sphingosine and hydroxy 24∶0 fatty acid was thereby identified in fractions 4 and 5, sulfatide with phytosphingosine and hydroxy 24∶1 fatty acid in fraction 6, and sulfatide with phytosphingosine and hydroxy 16∶0 fatty acid in fraction 7 (data not shown).
